# Rethinking bioinformatics expertise in the era of artificial intelligence

**DOI:** 10.1038/s41746-026-02777-1

**Published:** 2026-05-25

**Authors:** Wilson Wen Bin Goh, Annikka Polster, Limsoon Wong, Marija Cvijovic

**Affiliations:** 1 Lee Kong Chian School of Medicine, Nanyang Technological University, 59 Nanyang Drive, Singapore, Singapore; 2 Center for Biomedical Informatics, Nanyang Technological University, 59 Nanyang Drive, Singapore, Singapore; 3 School of Biological Sciences, Nanyang Technological University, 60 Nanyang Drive, Singapore, Singapore; 4 Center of AI in Medicine, Nanyang Technological University, 59 Nanyang Drive, Singapore, Singapore; 5 Division of Neurology, Department of Brain Sciences, Faculty of Medicine, Imperial College London, Du Cane Road, London, UK; 6 Institute of Mental Health, 10 Buangkok View, Buangkok Green Medical Park, Singapore, Singapore; 7 Chalmers University of Technology, Department of Life, Gothenburg, Sweden; 8 Oslo University Hospital, Oslo, Norway; 9 School of Computing, National University of Singapore, 13 Computing Drive, Singapore, Singapore; 10 University of Gothenburg, Department of Mathematical Sciences, Gothenburg, Sweden; 11 Chalmers University of Technology, Department of Mathematical Sciences, Gothenburg, Sweden

**Keywords:** Computational biology and bioinformatics, Mathematics and computing, Psychology, Psychology, Scientific community

## Abstract

Artificial intelligence (AI) is often framed as replacing scientific expertise, especially in bioinformatics. We argue instead that AI is a powerful accelerant whose value depends on expert guidance in design, data curation, interpretation, and governance. Because AI cannot judge biological meaning or verify scientific validity, bioinformaticians remain essential. Their role is shifting from workflow execution toward AI design, complex discovery, and responsible institutional leadership across research, translation, and clinical practice.

## Introduction

The dominant narrative surrounding artificial intelligence and scientific expertise is one of replacement^1–3^. Algorithms, the argument goes, are consuming the tasks that once required years of training, from writing code, interpreting data, synthesising literature, to generating hypotheses, and the professionals who built careers on those tasks are running out of time to adapt. In bioinformatics, this narrative has particular force. If a large language model can draft an RNA-seq pipeline, annotate variants, and produce a publication-ready interpretation of a differential expression analysis, what exactly is left for the bioinformatician to do?

The answer is: nearly everything that matters. And understanding why requires a clearer account of what artificial intelligence (AI) actually does, and what it cannot do without expert guidance.

The term ‘artificial intelligence’ encompasses a broad and heterogeneous set of computational approaches that differ substantially in how they work, what they require, and where they fail. For the purposes of this paper, we distinguish three overlapping categories relevant to bioinformatics. First, large language models (LLMs) and generative AI systems, including tools such as GPT-4 and Gemini, which produce text, code, and structured outputs by learning statistical patterns across vast quantities; these are the systems primarily responsible for the replacement narrative we address. Second, task-specific deep learning architectures — exemplified by AlphaFold2 and AlphaFold3^4,5^, which are trained on domain-specific datasets to solve precisely specified prediction problems; these represent a different mode of AI success, one driven by the quality of curated training data and biological domain knowledge rather than general-purpose generation. Third, agentic and hybrid systems, such as the Virtual Lab^6^, which orchestrate multiple AI components, tools, and reasoning steps toward complex scientific goals. The expert requirements, failure modes, and governance implications of these three categories differ considerably, and we return to these distinctions throughout.

AI does not understand biology. It recognises patterns in data it has been trained on and generates outputs that are statistically consistent with those patterns. This is genuinely powerful and should not be underestimated. But it also means that the quality of the AI output is only as good as the expertise brought to bear on it, in designing the analysis, curating the data, interpreting the results, and recognising when something that looks correct is, in fact, wrong.

Without that fundamental expertise, AI does not democratise science. It merely democratises the appearance of science^7–9^. This can manifest as analyses that look rigorous, outputs that sound authoritative, and conclusions that cannot be evaluated by the people who act on them. That is a different and considerably more dangerous thing than replacement.

Consider the most commonly cited example of AI’s incursion into bioinformatics: code generation. It is true that a large language model can produce a functional Python script for variant annotation, differential expression analysis, or genome assembly from a plain-language description^10,11^. What is less often acknowledged is that if you do not understand variant annotation, differential expression, or genome assembly, you cannot evaluate whether that script is doing the right thing. You cannot know whether it is using the correct reference genome, whether the statistical model is appropriate for your experimental design, whether the output is biologically meaningful or an artefact of a misspecified parameter. The code exists. However, the science, the capacity to judge whether the code is producing valid, interpretable, trustworthy results, does not come with it. It must be brought to the analysis by someone who knows what they are doing.

The distinction is worth stating plainly: AI is an extraordinarily powerful accelerant whose value scales directly with the expertise brought to bear on it. It is a springboard, not a replacement. It compresses the time required to execute analyses that experts have designed, validates approaches that experts have specified, and scales workflows that experts have validated. But it does not replace the expert judgement that makes those analyses worth running in the first place. The bioinformatician who understands this and who positions themselves accordingly is not threatened by AI. They become the essential condition for AI to produce cutting-edge science rapidly rather than noise.

This perspective lays out what that positioning requires, organised around three transitions: from running analyses to designing the intelligence that runs them; from executing established workflows to pioneering the complex, expert-dependent applications that generic AI cannot address; and from scientific contributor to institutional leader who ensures that AI is deployed responsibly, at scale, and in ways that can reach the clinic and the patient.

## Building the foundations: expert guidance as the condition for reliable AI

The first transition is technical, and it begins with a reorientation in how bioinformaticians think about their role. The ideal shift is from “AI consumer” to “AI custodian”. That is, from using AI to produce outputs to being personally responsible for whether those outputs are correct, reproducible, and biologically meaningful. This distinction matters because, without it, the most likely outcome of AI adoption in bioinformatics is not progress but rather a proliferation of noise in the guise of confident-looking analyses that nobody is equipped to critically evaluate.

## Designing the instructions that determine what AI produces

Prompt engineering, the design of instructions that guide LLMs toward accurate, relevant, and reproducible outputs, is now deemed a core scientific competency, with many institutions incorporating it into their undergraduate core curriculum^12^. This is not widely appreciated, but the implications (and ramifications) are significant. The same model, given differently constructed prompts, can produce outputs that range from biologically insightful to biologically nonsensical. The difference is not in the AI. It is in the expertise of the person directing it. This has been demonstrated empirically: Benchmarking studies show performance variation of 20-40 percentage points on identical biological tasks across different prompt formulations^13,14^, and performance that appears strong on standard benchmarks frequently fails to generalise to domain-specific biological questions not represented in the benchmark suite. Evaluation of such models requires entirely new strategies beyond simple performance measures^15,16^.

This creates both responsibility and opportunity for bioinformaticians: A prompt that reliably extracts pathway enrichment summaries from gene sets or consistently generates well-structured, statistically appropriate methods text from an analysis log is a reusable scientific asset. It encodes expert knowledge about what a good prompt input and LLM output looks like, and embeds deep insights that will remain invisible to non-domain experts who cannot differentiate a well-formed biological interpretation from a plausible-sounding one. Designing robust expert-curated prompt libraries for specific biological tasks, benchmarking outputs systematically across diverse inputs, and establishing those standards for the community to use and improve are opportunistic contributions that extend expert influence across every analysis that follows. However, this also means bioinformaticians must uphold responsibility as AI custodians, ensuring their stakeholders receive best practices and robust protocols to exploit AI safety correctly.

Combining expertise with more sophisticated prompt architectures further multiplies the bioinformation and AI synergy. Chain-of-Thought (CoT) prompting, where the model is instructed to reason step by step before producing an answer, substantially improves performance on complex biological reasoning tasks^13^, but only when the prompting strategy is designed by someone who understands what good biological reasoning looks like. Few-shot prompting, where carefully chosen biological examples are embedded in the prompt, can guide models toward domain-appropriate outputs, but choosing those examples requires exactly the domain knowledge the prompt is meant to convey. Thus, a proficient expert is not eliminated by these techniques. Synergising with AI effectively could considerably extend the bioinformatician’s reach and impact.

However, we must state that two caveats apply to prompt engineering as a scientific practice. First, chain-of-thought prompting^17^, despite its documented benefits for complex reasoning tasks, can be fragile: it can produce step-by-step reasoning that appears coherent but ultimately leads towards incorrect conclusions, while its performance varies substantially across AI models and tasks^13^. The second caveat, which is more fundamental, holds that prompt libraries and benchmarked outputs provide no guarantee of continued performance after foundation models are retrained. A prompt that reliably produces high-quality pathway enrichment summaries today may behave differently after the next model update. Hence, it is important to continuously monitor model drifts and uptake protocols/frameworks accordingly. This is not an argument against building prompt libraries; it simply means that we should treat them as living methodological assets that require ongoing validation and pairing them with the timely domain expertise needed to detect when outputs have silently degraded. This is not unlike post-deployment continuous monitoring mechanisms that have also been spelled out in clinical applications^18,19^.

Prompt engineering, however, should be understood as only one component of a broader design space rather than the ceiling of what AI tool development requires. Agentic systems, architectures in which multiple AI components are orchestrated to perform environmental perception, multi-step reasoning, tool use, memory retrieval, and iterative self-correction, represent an even more powerful and increasingly accessible paradigm^6^. The Virtual Lab illustrates this potential well: a human researcher defines a high-level scientific goal, and a coordinated team of LLM agents, each specialised in immunology, computational biology, or machine learning, designs and executes a pipeline incorporating ESM, AlphaFold-Multimer, and Rosetta to produce experimentally validated nanobody candidates (panel of experts)^6^. The expertise required to build, validate, and critically interpret such a system extends well beyond prompt construction. It includes understanding how to architect agent roles and responsibilities, how to design validation checkpoints that catch errors before they propagate, and how to interpret results in light of the specific limitations of each component model. The bioinformatician who can do this is not merely a prompt writer. They are effectively acting as AI engineers, infusing their deep domain knowledge into agents as proxied representations of their know-how, exactly the combination this paper argues is irreplaceable.

*In practice: treat prompts as living methodological assets. Version-control them, test them systematically across inputs, and document how outputs vary. A prompt library for five recurring tasks: variant annotation summaries, differential expression interpretation, methods drafting, literature synthesis, and figure legends, benchmarked across diverse inputs and published as a methods note, is a citable contribution to reproducible AI-assisted science*.

## Using automation to elevate, not replace, scientific thinking

Foundation models can now substantially automate a wide range of routine bioinformatics tasks: documentation, protocol drafting, iterative code generation, quality control scripting, variant annotation and results formatting^20^. This is genuinely valuable. But its value is conditional on having experts who can define what ‘routine’ means, specify the parameters within which automation is appropriate, and recognise when an automated output has gone wrong.

The GWAS pipeline illustrates this clearly. AI can generate and debug the scaffolding code for quality control, population stratification, and results formatting, tasks that are conceptually routine but technically time-consuming. What it cannot do is decide which QC thresholds are appropriate for this specific cohort and platform, recognise that a cluster of unexpected associations near a known locus may reflect linkage disequilibrium rather than an independent signal, or judge whether the population structure correction is adequate given the ancestry composition of the samples. These decisions require a bioinformatician to infuse domain expertise. The AI accelerates the analysis; the expert determines whether the analysis is valid. The consequences of inadequate expert oversight are not hypothetical. Failure to correctly account for population structure has historically produced false-positive associations that entered the literature and required subsequent retraction or correction^21,22^. AI-generated GWAS pipelines that automate stratification correction without expert verification of the population composition amplify this risk at scale.

The correct frame for thinking about AI-driven automation is that it should not be a replacement for scientific judgement, but as a way of concentrating it. When the mechanics of analysis are handled by AI, the bioinformatician’s attention is no longer diluted across scripting, debugging, and formatting. This frees up time and mind space to focus on valuable tasks such as experimental design, biological interpretation, methodological validation, and the pursuit of research questions that could not have been asked before the computational capacity to address them existed.

*In practice: identify the three most time-consuming routine tasks in your current workflow. Configure AI assistance for each, document the parameters and checks you apply to verify the outputs, and track how you use the recovered time. The recovered time is the metric. If it returns to the routine work streams in the same or other forms, then value-added transitions have not occurred*.

## Data quality: what AI learns from is what AI knows

One of the most consequential, and least visible, ways in which expert knowledge determines AI performance is through data. Model architecture receives most of the attention in AI research, but in practice, the quality, curation, and biological relevance of training data are at least as important, and often more so^23,24^. A model trained on data that is biased, mislabelled, or biologically uninformative will produce biased, mislabelled, or biologically uninformative outputs, regardless of its architectural sophistication. With LLMs, it will do so even more confidently, in a form that is indistinguishable from correct outputs to anyone who lacks the domain knowledge to evaluate them.

This has been demonstrated concretely in drug discovery, where addressing systematic issues in molecular representation, data quality, dataset size, and composition, rather than iterating on model architecture, produced improvements in accuracy and interpretability that rivalled deep neural networks using relatively simple machine learning approaches^23^. A systematic review of AI in drug discovery found that performance improvements attributed to model architecture frequently disappeared when training sets were curated to remove known data quality artefacts, suggesting that expert-driven data curation, not algorithmic sophistication, was the primary driver of reported gains^25^. The lesson generalises broadly: the expert’s ability to recognise a batch effect in a training set, identify label noise in clinical annotations, or flag that a particular molecular representation systematically disadvantages rare variants is not a secondary contribution to AI-driven research. It is often the primary one.

Treating data work as scientific work, investing in metadata standards, ontology alignment, provenance tracking, and systematic bias auditing, is therefore not administrative overhead. Data is the foundation on which trustworthy AI is built. Institutions that develop this infrastructure consistently outperform those that invest only in models, because they do not repeatedly solve the same upstream data problems in each new project. The bioinformatician who leads this work is not doing less science. They are making more reliable science possible for everyone who uses the resulting models.

*In practice: before any AI-assisted analysis, run a structured data audit. Check for batch effects, class imbalance, missing metadata, and annotation inconsistency. Document findings and corrections. This audit log belongs in the Methods section, and it is what distinguishes a rigorous analysis from one that has outsourced its quality control to a model incapable of performing it*.

## Explainability: the expert as the last line of defence

The opacity of current foundation models is not merely a technical inconvenience. In biomedical research, it is a scientific and clinical risk. A model that predicts drug response, identifies disease subtypes, or prioritises genomic variants with high apparent accuracy but no interpretable reasoning is not a scientific tool. It is a pattern-matcher whose outputs cannot be validated, challenged, or built upon in the way that scientific claims must be. And in a clinical context, it is a liability.

Current explainable AI (XAI) methods, including attention-based explanations, SHAP values, and feature importance scores, have well-documented limitations: they can be unstable across runs, sensitive to implementation choices, and in some cases represent post-hoc rationalisations of model behaviour rather than genuine explanations of it^26–28^. However, this in itself does not mean that these methods are inadequate and that XAI is a futile endeavour. Instead, their inadequacy is precisely what makes expert biological judgement indispensable. The bioinformatics expert’s role is not to simply accept XAI outputs as explanations. It is to interrogate them, to test whether the features a model attends to are biologically coherent, whether the reasoning holds under perturbation, and whether the explanation is consistent with what is known about the underlying biology. XAI in a biological context is more than just producing attention maps or feature importance scores. It means ensuring that the features a model relies on are biologically coherent, that a gene expression classifier is attending to genes with mechanistic relevance to the phenotype, not to housekeeping genes correlated with a batch effect. It means designing validation experiments that test whether inferred relationships hold under perturbation, not just in held-out samples from the same cohort. It means recognising that a model performing well on an internal benchmark may fail entirely on an external dataset and that this failure, if it happens after clinical deployment, has consequences that extend far beyond a retraction^26^.

Uncertainty quantification is an underutilised safeguard in the XAI space. A model that returns a calibrated confidence interval alongside a prediction, and that flags when a new input lies outside its training distribution, is far more useful in a scientific or clinical context than one that produces point estimates with apparent precision^29–31^. Building uncertainty estimation into pipelines and training clinical and research collaborators to interpret it requires exactly the combination of statistical sophistication and biological domain knowledge that defines the bioinformatician’s expertise. No amount of prompting can substitute it.

The practical implication is this: XAI methods should be treated as starting points for expert investigation, not endpoints. A feature importance score does not explain why a gene is important; it identifies a candidate for the expert to evaluate. An attention map does not validate a model; it can support in generating hypotheses that must be verified or tested. The expert is not a consumer of explanations that XAI produces. They are the source of the only explanations that ultimately matter.

*In practice: establish a minimum explainability standard before any model result is acted upon. This means: identifying the top predictive features and verifying they are biologically plausible; testing performance on at least one external cohort; and reporting a confidence or uncertainty metric alongside every prediction. These steps are not bureaucratic. They are what separates a scientific finding from a statistical artefact dressed up as one*.

## Driving discovery: the problems that require experts to be solved

The second transition is scientific. It requires bioinformaticians to identify the problems where AI is most powerful and where, without expert guidance, it is most likely to produce results that are wrong in ways that are difficult to detect. These are not the problems that lie at the margins of the field. They are its most important ones.

## Multimodal integration: where complexity defeats generic tools

The most consequential questions in contemporary biomedicine sit at the intersection of multiple data modalities: genomics and clinical records, proteomic profiles and imaging data, molecular signatures and longitudinal patient trajectories. Integrating these heterogeneous datasets, each with its own scale, resolution, noise structure, and biological logic, is arguably the most technically and conceptually demanding challenge in the field. It is also precisely the challenge for which generic AI tools are least adequate and expert guidance is most indispensable.

The difficulty is not computational. Algorithms for multi-omics integration, co-embedding, and cross-modal learning exist and are increasingly powerful. The issue is interpretation. When a joint model of genomic and clinical data identifies disease subtypes, the first question is not whether the clusters are statistically robust. It is whether they are biologically real, whether they reflect distinct molecular mechanisms, whether they align with clinical outcomes in a way that is actionable, and whether the features driving the separation are meaningful signals or technical artefacts. Answering these questions requires the kind of integrative expertise that comes from years of working at the boundary between data science and biology. A non-expert user of the same tool will produce outputs nonetheless, but they will not know whether those outputs mean anything.

The interpretive challenge of multimodal integration is compounded by a vocabulary gap that is rarely acknowledged but persistently consequential^32^. Technical communities tend to describe data in terms of platforms, events, schemas, and pipelines, a generic infrastructure language that abstracts away the core of experimental and biological context. Conversely, clinical and life sciences communities describe the same underlying data, focusing on regulated assays, experimental conditions, patient-level evidence, and clinical provenance, a language in which the context is the meaning. These are not merely stylistic differences. They reflect different mental models of what data is and what it is for. A ‘feature’ in a machine learning pipeline and a ‘biomarker’ in a clinical context may refer to the same measurement, but they carry different assumptions about how the measurement was obtained, what validates it, and what authorises its use in a decision^33,34^. Misalignment between these vocabularies is a consistent source of failures in cross-disciplinary AI projects: systems that are technically sound within one frame turn out to be clinically uninterpretable within another. The bioinformatician’s role as a translator between these worlds is therefore not peripheral to multimodal integration. It is central to it. The capacity to represent biomedical and clinical data requirements accurately to computational collaborators, and to explain computational outputs meaningfully to clinical ones, is as important as any algorithmic choice. It is also an emergent competency honed through hard-won experience working at the interdisciplinary interface rather than acquired from either side alone. Training programmes that place computational biologists in clinical environments, and clinicians in computational ones, are not a soft organisational preference. They are a precondition for the kind of integrated expertise that multimodal AI requires.

Designing co-embedding strategies that allow clinical and molecular features to inform each other while preserving biological interpretability, building fusion frameworks that can be validated against experimental and clinical outcomes, and communicating the results to clinical collaborators in ways that support rather than mislead decision-making: these are architect-level contributions. They are also the foundation of the next generation of precision medicine, and they cannot be made without the expert at the centre of the analysis. With AI, bioinformaticians are empowered to make more meaningful trans-disciplinary scientific contributions by infusing their knowledge with the technical process of today’s tools.

*In practice: identify one multimodal dataset in your environment currently being analysed in silos. Before choosing a method, write a one-page framing document: what biological question is the integration meant to answer, what would a meaningful result look like, and what would distinguish a real signal from a technical artefact? That framing document is the expert contribution that makes the subsequent analysis interpretable*.

## High-complexity applications: where shallow engagement produces wrong answers

As AI becomes accessible to non-experts, the most important distinction in the field shifts from who can run an analysis to who can tell whether the analysis is right. This distinction matters most in applications where the biological complexity is high enough that incorrect results are not obviously incorrect, where a plausible-looking output can be confidently wrong, and only someone with deep domain knowledge will notice.

Protein structure prediction illustrates the point. AlphaFold3 is a remarkable tool, and its outputs are widely used^4^. But the scientific value of those outputs depends entirely on the expertise brought to their interpretation. Which protein families are likely to have predictions of limited reliability? How should confidence scores be interpreted in the context of intrinsically disordered regions? How can predicted structures be used to prioritise mutagenesis experiments rather than simply to generate figures? These are not questions the model answers. These are questions the expert must bring to it^35,36^. A domain expert who understands the limitations of the predictions extracts qualitatively different scientific value from AlphaFold3 than a user who submits sequences and accepts outputs. The tool is the same, but the science is not.

The same principle holds across frontier applications: foundation models applied to gene regulation, single-cell trajectory analysis, three-dimensional genome organisation, or the interpretation of non-coding variants. In each of these use cases, the leading models are powerful while the failure modes are subtle. Recognising those failure modes, understanding what biological context is absent from a model’s training data, what confounders its architecture cannot distinguish, what experimental validation is required before a computational prediction becomes a scientific claim, becomes the expert’s irreplaceable contribution. It is also what makes the difference between results that advance the field and results that mislead it.

*In practice: for any high-complexity application you use, invest time in understanding where the model breaks down. Read the supplementary limitations sections of the original papers. Identify the failure cases. Then design your analysis or your validation strategy with those failure modes explicitly in mind. This is not scepticism about AI. It is the scientific rigour that makes AI-assisted results credible*.

## Transforming institutions: ensuring AI produces science, not the appearance of it

The third transition is organisational, and in many ways the most urgent. Scientific excellence at the level of individual analysis is necessary but not sufficient. The full risk of AI adoption in biomedicine is not that individual analyses will be wrong, though some will be. It is that AI will be deployed at institutional scale, in clinical and research settings, by people who are not equipped to evaluate its outputs, in ways that nobody has validated for the populations they will be used on. Preventing that outcome is a leadership responsibility, and it falls most naturally to bioinformaticians who understand the limitations of adopted AI models, can make data-informed strategic decisions, and sit at the intersections across both computational and biomedical disciplines.

## Closing the gap between proof of concept and practice

The history of bioinformatics is rich with powerful tools that never reach clinical practice. Not because they did not work, but because no one addressed the real-world conditions under which they could be used reliably and safely at scale. The gap between demonstrating that a method works in a controlled analysis and deploying it in a clinical workflow is not a technical gap. It is an implementation gap that requires attention to interoperability, user experience, documentation, stakeholder training, and regulatory alignment simultaneously^37^. It requires someone who understands both the science and the system into which it is being introduced.

A polygenic risk score for cardiovascular disease that performs well in a research cohort requires, before clinical deployment, integration with electronic health record systems, clinician training in how to interpret and communicate the score to patients, validation in the specific population it will be used in, and a process for updating it as evidence evolves. None of these is addressed by the original analysis^38^. All of them require cross-disciplinary thinking: technical, clinical, operational, and regulatory at once, which the bioinformatician is positioned to lead. Without that leadership, the tool stays in the paper. With it, it reaches the patient.

Adopting an implementation science mindset means asking, from the beginning of any project with translational potential, not only whether the method works but whether it can be used: by whom, under what conditions, with what training, and with what safeguards. It means building tools designed for the people who will use them, with documentation that a clinical collaborator can follow and outputs that a non-expert can interpret without misinterpretation. The AI systems that will transform healthcare are not necessarily the most technically sophisticated ones. They are the ones designed from the outset to be used correctly by people who are not their creators.

*In practice: the next time you complete an analysis with translational value, write a one-page implementation brief alongside the technical report. What would deployment at scale require? Who needs to be involved? What validation is still needed? What regulatory considerations apply? This brief will be more useful to a clinical or institutional partner than the methods section, and writing it will reveal what the real next steps are*.

## Ethics as scientific rigour, not regulatory compliance

The ethical risks of AI in biomedical research are, at their core, scientific risks. A diagnostic model trained predominantly on data from one demographic group will systematically underperform in others, a failure that is invisible to anyone evaluating it based on aggregate accuracy metrics alone. A clinical Natural Language Processing (NLP) system trained on historical records inherits the biases encoded in the language of those records, potentially perpetuating disparities in care. A genomic risk model deployed in a population that differs from its training cohort in ancestry, environment, or disease prevalence will produce predictions that are confidently wrong for the people who most need them to be right.

These are not edge cases or hypothetical concerns. They are documented failures that have occurred in deployed systems, and they share a common cause: the absence of expert oversight at the point where the model’s training data, architecture, and deployment context were defined^39,40^. Perhaps the most widely documented case is a commercial algorithm used to identify patients for additional care management, which was found to systematically underestimate the health needs of Black patients relative to White patients with equivalent disease burden^41^. The cause was a training objective that used healthcare costs as a proxy for health need, a design decision that encoded existing disparities into the model without any algorithmic error. Identifying and correcting this failure required exactly the combination of clinical domain knowledge, statistical scrutiny, and equity-aware evaluation that the algorithm’s developers had not initially applied. Bioinformaticians who understand the technical origins of these failures, who can audit a model for performance disparities across subgroups, identify the training data characteristics that produce them, and design governance protocols that prevent them, are not merely performing an ethical service separate from their scientific work. They are also doing science correctly.

This means proactively stratifying model performance by relevant subgroups before deployment, contributing to audit-trail frameworks that document training data provenance and known limitations, and engaging with regulatory standards such as GDPR and HIPAA, and the emerging landscape of AI-specific regulation, not as external constraints but as the codification of principles that rigorous science already demands. Bioinformaticians who lead this work do not simply protect institutions from liability. They protect patients from the consequences of AI that works well on average and fails for individuals.

The regulatory landscape for clinical AI has changed substantially since 2024. The EU AI Act (Regulation EU 2024/1689), the first comprehensive legal framework for AI globally, entered into force in August 2024 and classifies AI systems used in medical devices and clinical decision support as high-risk, subject to conformity assessment, transparency requirements, human oversight obligations, and post-market monitoring^42,43^. For AI systems embedded in regulated medical devices under EU MDR/IVDR, full compliance obligations apply from August 2027. Critically, the AI Act codifies in law many of the principles this paper argues for on scientific grounds: that high-risk AI systems must be developed with documented data governance, tested for performance across relevant subgroups, accompanied by adequate human oversight mechanisms, and subject to ongoing post-deployment monitoring. Bioinformaticians who lead the translation of AI tools from research to clinical practice are therefore operating in an environment where the scientific case for expert oversight and the legal obligation to provide it are now aligned. Familiarity with the Act’s requirements, and with analogous frameworks in other jurisdictions, including the FDA’s evolving guidance on AI-enabled medical devices^44^, is now a practical requirement for anyone designing AI systems intended for clinical use in Europe or in markets subject to European regulatory influence.

*In practice: add a bias audit to your standard analysis pipeline for any model intended for clinical or population health use. Stratify performance metrics by ancestry, age, sex, and relevant socioeconomic indicators. Report disparities explicitly. If the model underperforms in a subgroup, document it, flag it as a limitation, and treat it as an open scientific question. This is not additional work. It is the work*.

## Strategic leadership: the expert as institutional architect

The ultimate expression of the transition from tool user to AI architect is strategic leadership: the capacity to shape how institutions understand, invest in, and deploy AI across their research and clinical operations. This is where the full value of the bioinformatician’s cross-cutting expertise becomes visible: as the professional who can evaluate AI tools against biological and clinical requirements that vendors do not understand, who can design data infrastructure that serves scientific rather than commercial logic, and who can translate the implications of AI capabilities and limitations into language that department heads, clinical directors, and executive leaders can act on.

The trajectory is already visible across research-intensive health systems. As institutions grapple with how to govern, deploy, and extract value from AI at scale, they are increasingly turning to professionals who can bridge biological knowledge, computational fluency, and organisational complexity, a combination that describes the bioinformatician more precisely than it describes almost any other role. Appointments of computationally trained life scientists to positions such as Chief Data Scientist, AI Director, and national programme lead for data science research and training are becoming less exceptional and more indicative of a structural shift in how scientific institutions define leadership capability in the AI era^40^. The strategic questions that drive these appointments, such as which AI tools are worth investing in, how data infrastructure should be architected, how AI capabilities should be embedded in research and clinical workflows, and how staff should be trained, are questions that bioinformaticians are better equipped to answer than almost anyone else.

Getting there requires deliberate investment beyond technical skill: in the ability to communicate complex ideas to non-technical stakeholders, to manage resistance and build coalitions for change, and to construct and defend strategic recommendations. It also requires maintaining a genuinely current understanding of a field moving fast enough that last year’s capabilities are already obsolete. But for bioinformaticians who make this investment, the opportunity is not merely career advancement. It is the chance to determine whether AI in their institution produces reliable, ethical, clinically valuable science or whether it produces, at scale, the appearance of science without the substance.

*In practice: identify one institutional decision about AI currently being made without bioinformatics input, a vendor evaluation, a data infrastructure choice or a training programme design. Offer a structured perspective: a one-page briefing that frames the decision, outlines the key scientific and technical considerations, and makes a recommendation. Strategic influence in institutions is built exactly this way, like one well-timed, well-framed contribution at a time*.

## Conclusion

The conversation about AI and scientific expertise has been framed, almost universally, as a question of replacement: what will AI take over, how fast, and who will be left? This framing is not only unhelpful. It is wrong. It misunderstands what AI is, what it produces, and what is required to make its outputs scientifically valuable.

AI is genuinely capable of accelerating discovery from identifying patterns in data at a scale and speed no human analyst can match, surfacing candidate hypotheses from vast literature, and compressing the distance between raw data and interpretable results. But acceleration is not the same as validation. The transformation of that output into knowledge into findings that are valid, reproducible, interpretable, and clinically actionable, requires expert judgement at every stage of the process: in the design of the analysis, the curation of the data, the interpretation of the results, and the recognition of failure when it occurs. Remove the expert, and what remains is not science conducted more efficiently. It is the appearance of science, conducted at scale, by systems that cannot know when they are wrong. The risk is not merely inefficiency. It is the production, at institutional scale, of what looks like science and is in fact, noise: results that are superficially statistically coherent, computationally sophisticated, but scientifically unvalidated. AI creates that risk precisely because it is so capable of producing convincing outputs. Expertise is what ensures judicious use of AI to convert outputs into knowledge.

The three transitions we have described: from tool user to technical custodian, from pipeline executor to pioneer of expert-dependent discovery, from scientific contributor to institutional architect, are not accommodations to a threatening future. They describe what AI actually requires to work properly. Bioinformaticians who understand this are not running to keep pace with AI. They are custodians, providing indispensable conditions for AI to be useful rather than dangerous.

The bioinformatics profession is not becoming obsolete. But it is being asked a clarifying question: do you understand the science you are doing deeply enough to know when the machine is right and when it only looks that way? The answer to that question, more than any technical skill or tool proficiency, is what will define the bioinformatician’s role in the next decade of research and medicine.

The test has already begun. And it turns out that what is being examined is not whether you can use AI. It is whether you understand enough to be the expert behind it.

## Data Availability

No datasets were generated or analysed during the current study.
